# A Single-Trial P300 Detector Based on Symbolized EEG and Autoencoded-(1D)CNN to Improve ITR Performance in BCIs

**DOI:** 10.3390/s21123961

**Published:** 2021-06-08

**Authors:** Daniela De Venuto, Giovanni Mezzina

**Affiliations:** Department of Electrical and Information Engineering, Politecnico di Bari, Via E. Orabona, 4, 70124 Bari, Italy; giovanni.mezzina@poliba.it

**Keywords:** brain–computer interface (BCI), P300, single-trial detection, autoencoder, CNN

## Abstract

In this paper, we propose a breakthrough single-trial P300 detector that maximizes the information translate rate (ITR) of the brain–computer interface (BCI), keeping high recognition accuracy performance. The architecture, designed to improve the portability of the algorithm, demonstrated full implementability on a dedicated embedded platform. The proposed P300 detector is based on the combination of a novel pre-processing stage based on the EEG signals symbolization and an autoencoded convolutional neural network (CNN). The proposed system acquires data from only six EEG channels; thus, it treats them with a low-complexity preprocessing stage including baseline correction, windsorizing and symbolization. The symbolized EEG signals are then sent to an autoencoder model to emphasize those temporal features that can be meaningful for the following CNN stage. This latter consists of a seven-layer CNN, including a 1D convolutional layer and three dense ones. Two datasets have been analyzed to assess the algorithm performance: one from a P300 speller application in BCI competition III data and one from self-collected data during a fluid prototype car driving experiment. Experimental results on the P300 speller dataset showed that the proposed method achieves an average ITR (on two subjects) of 16.83 bits/min, outperforming by +5.75 bits/min the state-of-the-art for this parameter. Jointly with the speed increase, the recognition performance returned disruptive results in terms of the harmonic mean of precision and recall (F1-Score), which achieve 51.78 ± 6.24%. The same method used in the prototype car driving led to an ITR of ~33 bit/min with an F1-Score of 70.00% in a single-trial P300 detection context, allowing fluid usage of the BCI for driving purposes. The realized network has been validated on an STM32L4 microcontroller target, for complexity and implementation assessment. The implementation showed an overall resource occupation of 5.57% of the total available ROM, ~3% of the available RAM, requiring less than 3.5 ms to provide the classification outcome.

## 1. Introduction

Brain–computer interfaces (BCIs) are platforms able to integrate straightforward interactions between the human brain and the external world, “bridging” users’ brain signals directly to a mechatronic device without the need of any physical interaction [1]. BCIs were initially developed to help locked-in patients in formalizing specific requests [1]; nevertheless, in recent years, the BCIs’ versatility has also spread their use among healthy subjects in areas such as entertainment [2], car driving [3], smart homes [4], speller systems [5], mental state monitoring [6], Internet of Things applications [7], etc.

For these kind of applications, electroencephalogram (EEG)-based BCIs are the most diffused type due to their noninvasive nature, safety, and the inexpensiveness of the dedicated acquisition devices, compared with BCIs that exploit, for instance, electrocorticogram (ECoG) or functional near-infrared spectroscopy (fNIRS) [1,8,9,10,11].

There are several types of EEG patterns used in BCI applications, such as event-related potentials (ERPs), steady-state visual evoked potentials (SSVEPs) and motor imagery (MI). Specifically, the first type (i.e., ERPs) is the most used because of the simplicity of the elicitation paradigms implementation [8,11]. Moreover, since these potentials are time-locked with respect to the stimulation onset, they are optimal candidates for repetitive and reliable practical BCIs [11]. ERPs are characterized by positive and negative signal deflections, namely components, with each one being linked to different cognitive processes and elicitable with different paradigms. A particular ERP component that has found large use in the BCI area is the P300. Compared with other potentials, the P300 is simple to be elicited and measured, requires low training time with no complex paradigm and is suitable for most subjects, including those with severe neuromuscular diseases [1].

This work is focused on the P300 signal elicited during a classic odd-ball paradigm [8], in which a series of stimuli are presented to the subject. In general, these stimuli can be categorized into two types: frequent and rare ones. The occurrence of a rare stimulus causes the elicitation of the P300 component [8].

P300 ERPs typically present a very low signal-to-noise ratio (SNR) if considered in a single trial [9]. For this reason, in most BCI applications, several trials are stored and averaged over an increasing number of repetitions, in order to emphasize the time-locked components (e.g., P300) and minimize the background noise [10]. This grand-average method improves the robustness of the system but, at the same time, it drastically reduces the BCI speed in terms of information translate rate (ITR). For instance, considering a widely diffused P300 speller problem, ITR moves from an average value of ~11 bits/min [10,11,12,13] in a single repetition context, to ~8 bits/min after accumulating the 15 repetitions constituting the character epoch [8]. The issue can be mitigated by adaptive flashing times techniques [14]. However, the correct classification of the P300 response in a few repetitions is a crucial target for the design of fast BCI systems.

Several solutions have been proposed in the literature to address the single-trial P300 recognition problem. Most of them use traditional shallow learning for the P300 classification purpose. In this context, the authors in [13] filtered the EEG signals in the interval 0.1–20 Hz. The filtered signal is then decimated according to the high cut-off frequency. At this point, each signal from a given channel is characterized by 14 samples. Next, they divided the EEG trials into several data subsets, each one connected to a dedicated support vector machine (SVM), for a total of 17 SVMs. To work properly, the implemented algorithm requires a first channel selection stage based on a recursive channel elimination. The proposed system was demonstrated to achieve a P300 detection accuracy of 25.5% after a single repetition and 96% after 15 repetitions in the BCI competition III speller problem (i.e., selection among 36 characters). Authors in [15] also achieved similar results by implementing a Bayesian P300 detection method based on the maximum regression target probability value. Despite the good results, the method suffers from a very long computation time. Indeed, the method requires, on average, 7 min to complete the proposed task (four six-character words and a random number), against about 2.5 min required by most of the equivalent approaches [15]. A processing chain made up of a continuous wavelet transform (CWT), scalogram peak detection, and linear discriminant analysis (LDA) was used for this purpose by the authors in [16]. A misclassification error of 45% was reached by the method, which won the BCI competition II [16]. In the same shallow learning application context, authors in [17] investigated the possibility of exploiting (temporal) attention processing, widely adopted in SSVEP-based BCIs [18] and in EEG-based emotion recognition [19], to improve P300-based brain–computer interface (BCI) performance. Their study, typically oriented to amyotrophic lateral sclerosis (ALS) patients, demonstrated the investigation of those cognitive processes and using a stepwise linear discriminant analysis (SWLDA) for the offline classifier coefficient calibration and the identification of the character as the intersection between the row and the column exhibiting the maximum of the sum of scored features. In P300 speller context, by averaging all the waveforms from target and non-target trials composing each run (i.e., 15), the system in [17] demonstrated the ability to approach P300 state-of-the-art accuracy (i.e., 98%) by using only six channels. Nevertheless, the authors did not test the same system on the BCI competition Dataset III [13], making a possible comparison unsatisfying.

In the recent past, a growing interest has been dedicated to the deep learning application in P300 detection. Deep learning approaches strongly impacted natural language and computer vision processing, allowing the resolution of up to that moment still unsolved real-world problems [11]. Deep learning application for P300 detection is still in an evolving stage, far from a consolidated methodology [10].

The first authors who exploited the deep learning capability of capturing hierarchical features from EEG signals were Cecotti and Graser in their work [12]. Here, they implemented a four-layer convolutional neural network (CNN) to capture spatial and temporal features from the raw EEG signals in input. Seven different CNN architectures were proposed with three ensemble models. The most performant among the proposed methods was shown in the results reached by the authors in [13], i.e., 25.5% after a single repetition and ~95% after 15 repetitions in the speller problem. This approach was exploited by the authors in [10], which extended the model by introducing a batch normalization layer to prevent CNN overfitting. The proposed method, named BN3, analyzed EEG data as a bidimensional matrix with the {channels × time} shape, acting on it as an image structure and reaching the same results of the authors in [13] after 15 repetitions, with an improvement of +10% of character accuracy on a single repetition. A similar approach was adopted by the authors in [20], who—differently from [10]—analyzed an EEG trial as a 3D matrix. Their work exploits spectral features from three different frequency bands (i.e., θ, α, β) creating from each time instant an RGB image given by the linked spectrogram. Then, they stacked convolutional layers with a long short-term memory (LSTM) neural network, increasing the system complexity without any substantial improvements against the above-listed state-of-the-art results. Similar results were achieved in [21], where a 3D recurrent neural network (3DRNN) was used to detect a P300 signal in a single trial. Finally, authors in [22] proposed the introduction of a preprocessing stage based on principal component analysis (PCA) to improve the CNN results. The proposed system demonstrated a slight improvement of the results, starting from eight repetitions up to 15. Recently, autoencoder neural networks are also approaching the brain activity analysis field thanks to their capabilities in denoising the EEG signal. Autoencoders impacted on the seizure detection field [23] realizing a useful feature extractor step, if empowered with CNNs and LSTMs in the encoder–decoder setup. Authors in [24] exploited this concept, proposing an event-related potential encoder network (ERPENet) that incorporates four 2D CNNs and two 512-units LSTMs in the autoencoder setup structure, trying to simultaneously compress the input EEG signal and extract-related P300 features into a latent vector. This vector was sent to a single one-unit fully connected layer for classification. The model provided a single-trial accuracy of 83.54% (with an area under curve of 69%) on the P300 speller problem included in the BCI competition III dataset, analyzing 35 channels from the central-parietal lobe.

In this framework, this paper proposes the design, implementation and test of a novel architecture for single-trial P300 detection with two main aims: (i) maximizing the ITR, while keeping high recognition accuracy for a low number of stimuli repetition and (ii) ensuring the full system implementability on a low-cost dedicated microcontroller-based platform. The first design focus allows the BCI, which exploits the here presented P300 detector, to increase its speed, ensuring a human–machine interaction that is as fluid as possible. It represents a critical constraint for those BCI in which the neural interface speed can strongly impact on the safety of the system, such as in P300-based car driving [3], or in which a BCI becomes an important enabling technology, such as in the case of P300-based word speller for people with disabilities [10,11]. To ensure this condition, in this paper, a punctual inference timing analysis has been carried out. It permitted the identification and the correction of all the time-critical parts in the architecture. The second design focus, which represent a novelty in the NN architecture design workflow, concerns the architecture implementability analysis on a widely diffused family of low-cost microcontrollers. This analysis provides a good starting point for the conceptualization and the realization of a dedicated low-cost and mobile P300-based BCI. In particular, by keeping the consumption of resources of the microcontroller far below their availability, the design of a dedicated P300-based BCI board is also considerably simplified, with positive consequences on board footprint (e.g., external memories and related circuitry no longer needed) and power consumption (e.g., no external read/write operations, only microcontroller static/dynamic consumption).

Keeping in mind the above, the here-proposed P300 detector bases its working on the combination of a novel pre-processing stage based on the EEG signals’ symbolization and a first parallel autoencoding stage followed by batch-normalized CNN for the temporal filtering. We refer to this architecture as an autoencoded CNN in the following. Specifically, the proposed system acquires data by six EEG electrodes from central and parietal brain lobes. Next, it submits EEG signals to a first low-complexity preprocessing stage including low pass filtering, baseline correction, windsorizing and symbolization. The symbolized EEG signals are then sent to an autoencoder model to emphasize those temporal features that can be meaningful for the following CNN stage. Autoencoded EEGs are then concatenated and used to feed a seven-layer network, which includes a 1D convolutional layer and three fully connected layers. Inputs, convolutional and fully connected layers are preceded by a batch normalization. The realized network is then validated on an STM32L4 microcontroller target, for complexity and implementation assessment. Since all of the analyzed state-of-the-art studies use the Farwell and Dochin P300-based speller as a testbench [8] to compare among each other their system capabilities, in this paper, two datasets are analyzed to assess the algorithm performance: (i) BCI competition III data from a P300 speller testbench and (ii) self-collected data from a prototype car driving experiment.

Experimental results from the first dataset, i.e., P300 speller one, showed that the system is able to outperform the state of the art in terms of harmonic mean of precision and recall (F1-Score = 51.78 ± 6.24%), ensuring a +5 bits/min of average ITR if compared with other state-of-the-art solutions. The same method, applied to the prototype car driving experiment, provided an F1-score of 70.00% and an ITR > 30 bit/min, allowing a car direction change every 1.8 s. The implementation feasibility assessment on an STM32L4 microcontroller showed that the overall system occupies 5.57% of the total available ROM, ~3% of the available RAM.

The paper is organized as follows: Section 2 describes the P300 paradigms, detailing the considered datasets, the preprocessing stages and the model architecture. Section 3 and Section 4 are dedicated to the experimental results and their discussion, respectively, while Section 5 concludes the paper, underlining the achievements and perspectives. 

## 2. The Method

### 2.1. Datasets and Stimulation Protocols

**Datasets.** Two datasets were investigated and described in the following. Both of them shared the same processing chain but differed among each other for the final application and the acquisition device. One dataset, namely Dataset 1 in the following, was related to data collected by the team of the DEISLab (Politecnico di Bari, Italy) on voluntary subjects in the context of prototype car driving [3]. The subjects group was composed of four students (healthy subjects) recruited to participate in this BCI experiment. The second dataset, chosen for comparison purposes and named Dataset 2 in the following, was a dataset provided by the Wadsworth Research Center NYS Department of Health for the international BCI competition III [25]. This dataset collected EEG data from two subjects who participated in the competition.

More in detail, Dataset 1 contained trials acquired by a 32-channel wireless EEG headset by g.Tec (i.e., g.Nautilus Research), considering AFz as a reference channel and A2 (right ear lobe) as a ground lead. Specifically, the monitoring for this application was focused on 6 specific channels, i.e., Fz, Cz, Pz, PO7, Oz, PO8, resulting from a statistical offline recursive channel elimination routine all over the four subjects, as better explained in Section 2.3, and confirmed by the best electrodes configuration for P300 speller applications, found by the authors in [26].

EEG data were acquired with a resolution of 24 bits and transmitted at 250 Sa/s, reduced to match the 240 Sa/s from Dataset 2. The EEG signals were band passed in the interval 0.1–20 Hz before the transmission. 

The second dataset [25] was composed of EEG trials collected by 64 channels using the BCI2000 system [25]. The monitoring was focused, also in this case, on 6 selected channels to match the architecture input size. Differently from the Dataset 1, the analyzed EEG channels from the two involved subjects statistically answered in different ways to the recursive channel elimination routine (Section 2.3). For the first involved subject (namely subject A in the following), the six selected channels were: FCz, C2, CP5, CPz, P6 and PO7, while for the second subject, similarly named subject B, the electrodes CPz, PO7, POz, PO4, PO1, O1 were chosen. Signals from the selected channels were sampled at 240 Hz and bandpass filtered into 0.1–60 Hz. 

**Figure 1:**
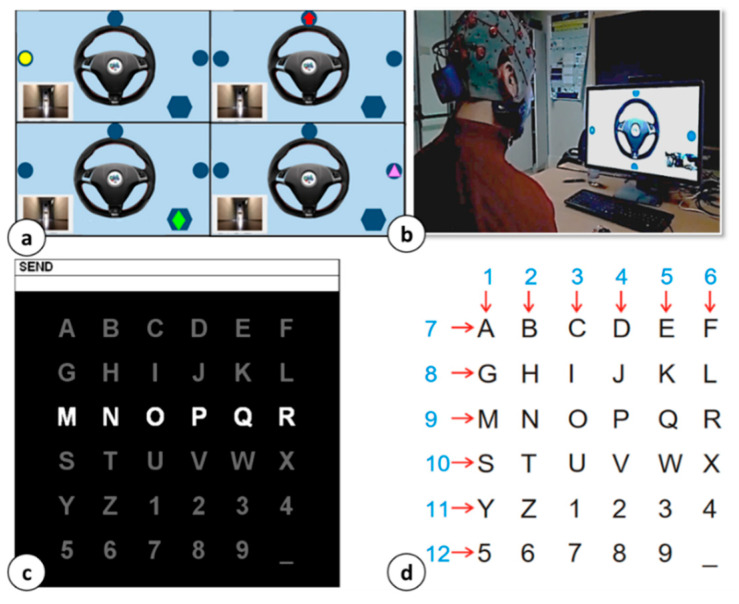
BCI stimulation protocol. (**a**) Snapshot of the 4 targets composing the prototype car driving odd-ball paradigm; (**b**) experimental setup for the prototype car test; (**c**) 6 × 6 character matrix for P300 speller diagram from BCI competition III; (**d**) row and columns indexes distribution.

**Stimulation Protocols.** For Dataset 1 data collection, the subjects underwent the 4-choice paradigm, already proposed in our previous works [3,23] and reported in Figure 1a. The protocol, designed according to the odd-ball paradigm, consisted of four visual stimuli, individually and randomly flashing on a display (25% occurrence probability) with an inter-stimuli time (ISI) of 200 ms. Each stimulus persisted on the screen for 100 ms. When the user focused their attention on a particular stimulus (so they are expressing a direction), the stimulation could be considered a classical binary discrimination problem. As reported in [3], the above-described protocol can be easily used to drive a prototype car. A snapshot of the experimental setup for the protocol is shown in Figure 1b. For the training set, a total of 1520 target trials (with elicited P300) and 4560 non-target trials (without P300 contributions) were considered per each involved subject. The test set was, instead, composed of 500 target trials and 1500 non-target trials per subject. The subjects involved in the BCI experiments are numbered from 1 to 4 in the following. The composition of the presented dataset is summarized in Table 1.

Data composing Dataset 2 were collected considering—as stimulation paradigm—the 6 × 6 characters matrix in Figure 1b. During the competition experiments, each of the 12 between rows and columns flashed randomly with an ISI of 175 ms and a flashing duration of 100 ms. The subjects were asked to focus on a specific character. Each row and column were flashed 15 times, constituting a character epoch. The selected character prediction was typically generated at the end of each character epoch. For the training set, 85 characters per subject were considered, for a total (per subject) of 2550 target trials and 12,750 non-target trials. The test set was composed of 100 characters for a total of 3000 target trials and 15,000 non-target ones. The subjects involved in the BCI experiments are identified by A and B in the following, according to the BCI competition nomenclature. Table 1 summarizes the training and test datasets composition.

### 2.2. The Architecture

**Figure 2:**
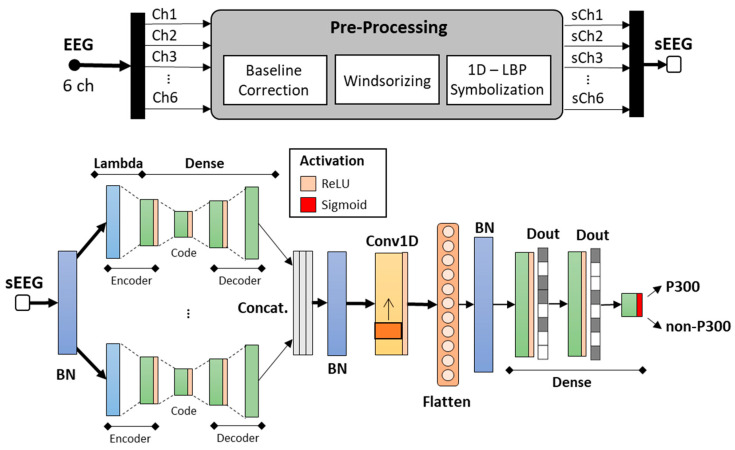
Overall architecture overview.

The network architecture of the proposed single-trial P300 detector is schematically depicted in Figure 2. The BCI workflow, shown in Figure 2, can be summarized as follows. First, the EEG signals from the acquisition device (or from the offline test dataset in Dataset 2) underwent a preprocessing stage. This stage consisted of three main steps: (i) baseline correction, (ii) windsorizing and (iii) local binary patterning (LBP) symbolization. An optional additive bandpass filtering stage should be included before all the above-listed ones, if not already embedded in the acquisition device (e.g., g.Nautilus Research signal conditioner includes this stage before the transmission). 

The first optional stage required a bandpass filter with high and low pass cutoff frequencies of 0.1 Hz and 20 Hz, respectively. For the sake of repeatability, the filter utilized for the Dataset 2 corresponded to the 8th order Butterworth included in the g.Nautilus Research device. The filtered EEGs were then submitted to a trial extraction stage in which a specific time window after the stimulus onset was selected. Next, the trials were treated by a local baseline correction routine, which consisted of aligning each trial around a “zeroed” initial point. The corrected EEG trials were analyzed by a windsorizing procedure that truncated signal outliers, giving them a predefined value. Finally, the windsorized signals went through a last step preprocessing step: the LBP symbolization. This routine, widely known in the image-processing field, permitted one to analytically transform experimental measurements such as 1D-time-series into a series of binary strings. As it will become clearer shortly, this step generated a signal per channel that ranged between 0 and 8 with single unit discrete steps [27].

The symbolized EEG signals (sEEG in Figure 2) became the input for the proposed autoencoded CNN. The proposed neural network, expanded at the bottom of Figure 2, consists of a total of 13 layers, namely L_0~13_ in the following. 

The NN embedded a first batch normalization (limited to the training activity adaption), then it implemented a first tensor slice on the six selected EEG channels (Lambda in Figure 2). Each sliced channel went through an autoencoder based on 3 fully connected layers, with rectified linear unit (ReLU) activation function, which represented—in the order—the encoder, the code, and the decoder of the autoencoder architecture. The last layer consisted of a dense layer with the same size as the sliced channel in input, with a linear activation function (not explicitly reported in the model).

The six outcomes were then concatenated in a tensor with size {timesteps, channels}. Next, the tensor fed a 1D convolutional and subsampling layer for temporal feature extraction. ReLU activation was also used in this case. The convolutional layer outcome was then flattened and sent to a deep network made up of 2 dense layers, preceded by a batch normalization step to avoid distribution shift, which led to signal saturation, decelerating the learning [28]. During the training, both the dense layers composing the deep NN were supported by a dropout operation to reduce the overfitting phenomenon and favor the system generalization. The architecture ended with a single-unit dense layer and a sigmoid activation that returned a value between 0 and 1. It returned the probability that the specific sample is related to a P300 trial or a non-P300 one. A threshold system ensured the binary classification.

### 2.3. Recursive Channels Elimination

Aiming to reduce the memory usage and to favor the overall system implementability, a first dimensionality reduction problem on the input size was needed. Considering—for instance—the g.Nautilus Research solution presented in Section 2.1 for the Dataset 1 data collection, an input matrix of 24 bits/sample × 32 channels × 250 samples/s should be analyzed every second (for a total of 24 kB). In a similar way, considering the BCI2000 system used for the Dataset 2 composition, an input matrix of 24 bits/sample × 64 channels × 240 samples/s, for a total of 46.08 kB, would become the input matrix for the overall system. Considering a parallel autoencoded CNN implementation as the one proposed in the Section 2.2, it would result in a strong increment of the network complexity and, thus, of resources usage. For this reason, an offline recursive channels elimination step preceded the nominal working of the architecture. This processing step’s role was to find a 6-channel combination (24 bits/sample × 7 channels × 250 samples/s for an input flow of 5.25 kB/s) able to ensure the best channel selection criterion value proposed by the authors in [13]. It is mathematically defined as:

(1) $$Criterion=\frac{\mathrm{TP}}{\mathrm{TP}+\mathrm{FP}+\mathrm{FN}}$$

where TP is the number of P300 trials correctly classified, TN is the number of non-P300 trials correctly classified, FP is the number of non-P300 trials misclassified (classified as P300 trials) and, similarly, FN is the number of P300 trials misclassified.

For the recursive channel elimination purpose, a provisional NN architecture model was instantiated. This provisional NN was inspired by the BN3 one proposed in [10] but discarded the spatial filtering layer (i.e., the first one). This architecture was chosen because of its capability to return promising results only by analyzing raw EEG signals. All the combinations were tested on the provisional neural network considering the training set. Specifically, a validation split on 30% of the training dataset was considered in a holdout validation setting and the validation criterion value for each combination was extracted.

Firstly, the routine started with all the 32 (Dataset 1) or 64 channels (Dataset 2); then, each channel was eliminated, and the criterion score was computed on the remaining 31 (Dataset 1) or 63 (Dataset 1) channels. The procedure was repeated for all the involved channels. The channel, whose elimination led to the highest criterion score, was discarded. Next, the procedure was repeated up to finding the best 10 channels per subject. A statistical analysis was thus carried out to find the channels that are mostly shared among the involved subjects, aiming to favor the application generalization. Considering Dataset 1, the six channels that fell within the best ten per all the subjects were Fz, Cz, Pz, PO7, Oz, PO8. Differently, on the Dataset 2 training set, only the CPz and PO7 channel resulted in being shared among the two subjects. The remaining best channels for subject A resulted to be FCz, C2, CP5 and P6, while for the subject B, POz, PO4, PO1, O1. 

### 2.4. Data Preprocessing

Once the stimulation occurred (i.e., row/column or direction flash), the acquisition device started streaming data to a dedicated parametrizable depth buffer. This buffer depth defined the time window composing the trial. As introduced in Section 2.2, each trial underwent three main pre-processing steps: (i) baseline correction, (ii) windsorizing and (iii) LBP symbolization. This section discusses them in more detail, providing experimental hyperparameters choice.

**Baseline correction.** For the proper window definition of the local baseline correction, firstly, the same provisional NN architecture model presented in Section 2.3 was instantiated. Three different time windows for trials were then analyzed, changing the buffer size: (i) [0 s, 1 s]; (ii) [0 s, 0.8 s]; (iii) [0.1 s, 0.8 s]. For each of these time windows, two kinds of baseline extraction approaches were tried: (i) baseline calculated as the average of the first 50 ms of the time window or (ii) baseline calculated as the average of the first 100 ms of the time window. In an analog way to the procedure described in Section 2.2, all the combinations were tested on the provisional neural network considering the training set. Specifically, a validation split on 30% of the training dataset was considered in a holdout validation setting. The chosen assessment criterion was the harmonic mean of precision and recall, the F1-Score, which is defined as:

(2) $$F_{1}=\frac{2\mathrm{TP}}{2\mathrm{TP}+\mathrm{TN}+\mathrm{FP}+\mathrm{FN}}$$

A grid search routine among the analyzed combinations returned that the best window and, thus, the selected one was the range [0.1 s, 0.8 s] for a total of 168 samples, while the chosen baseline correction consisted of extracting the average in the interval [0.1 s, 0.15 s] for a total of 12 samples. The average value was then subtracted for all the trial values.

**Windsorizing.** Subject movements, eye movement, eye blinking, and muscle activity can cause large amplitude outliers in the EEG. To reduce the effect of these outliers, the data from each channel should be windsorized according to the preprocessing guidelines proposed for a P300-based BCI provided by the authors in [29]. As the first step, for each channel, the 1st and the 99th percentile, all over the training set, were computed. Next, all those amplitudes lower than the 1st percentile were replaced with the 1st percentile value. Similarly, all those amplitude values higher than the 90th percentile were assigned to the 99th percentile fixed value. the known percentile presence, in terms of trial length percentage, can represent a useful indicator for the most informative channels. For instance, it was observed that several trials (~100 P300 trials, ~200 non-P300 trials) in Dataset 2 were affected on the Fz channel by more than the 40% of winsorizing values. Optical inspection confirmed the presence of physiological and non-physiological artifacts on this channel [30].

**Figure 3:**
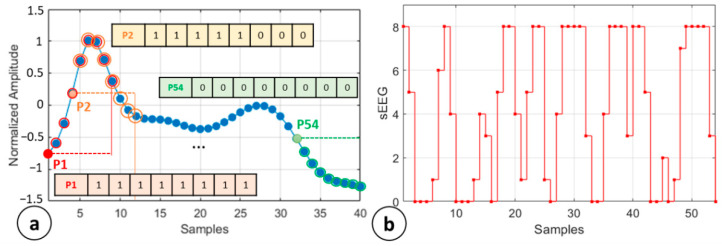
Implementation of 1D-LBP code. (**a**) 40-sample EEG data subset with LBP code extraction (**b**) sEEG from LBP routine application on a 168 samples trial.

**LBP symbolization.** The LBP operator was introduced in [31] as a powerful tool for the texture description and defined as a gray-scale invariant texture measure. In that application, for each pixel in a 2D image, a binary code is produced by considering a central pixel as a threshold and analyzing 8 neighbor pixels. This approach also found large application in treating experiments based on 1D-time series such as EEG analysis, especially in seizure detection [27]. In this paper, we propose the application of a custom LBP symbolization routine aiming to improve the network performance. The 1D-LBP method is described step-by-step by considering a segment of 40 samples from 168 sample EEG trial, as per Figure 3a. The LBP routine starts identifying all the onset points for the binary code assessment, namely P_1,…54_ in Figure 3a. These P samples have a range that goes from 1 to 160 (8 samples are left on the right of P_54_ to avoid index overflow) with step 3. Considering a comparison window of *n* = 8 samples, as shown in Figure 3a, the amplitude value related to the jth sample ($P_{j}$) is set as a local threshold and the following 8 samples are compared with it. The resulting LBP code is given by:

(3) $$\mathrm{sEEG}\left(k\right)=\sum_{i=P_{j}+1}^{P_{j}+n}\left(\left(\mathrm{EEG}\left(i\right)-\mathrm{EEG}\left(P_{j}\right)\right)>0\right)\;\;\mathrm{with}\;P_{j}=1:3:160$$

where sEEG(j) is the kth sample of the sEEG signal in Figure 3b, $\left(\mathrm{EEG}\left(i\right)-\mathrm{EEG}\left(P_{j}\right)\right)>0$ is a logic condition returning 1 if EEG(i) is higher than the EEG in the sample $P_{j}$, zero otherwise. The resulting sEEG signals consisted of 54 samples ranging between 0 and 8. The preprocessed data were then ready to be analyzed by the proposed autoencoded CNN.

### 2.5. Autoencoded-CNN: Network Topology

The here proposed network consists of 6 parallel 5-layer autoencoder structures followed by 6 sequential layers. 

**Figure 4:**
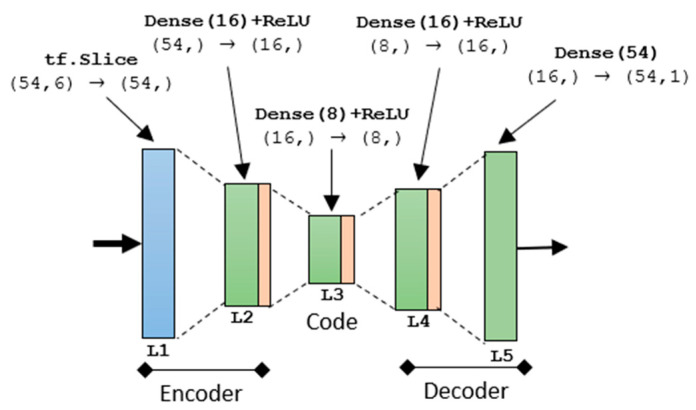
5-layer Autoencoder Implementation.

Specifically, the first layer L_0_ consists of a batch normalization layer. This layer aims to normalize the input data with respect to the mean and the standard deviation of the training batch. In the very first stage, it is necessary to feed the network with the proper data format. The resulting tensor, with size (54,6), is used to feed 6 parallel autoencoders, one per channel. The autoencoder network is the same for all 6 parallel branches and is reported in Figure 4. 

Autoencoders are neural networks with the aim of generating new data by first compressing the input into a space of latent variables and, subsequently, reconstructing the output based on the acquired information. This type of network consists of two parts: encoder and decoder. The code is placed in the middle of the coding–decoding procedure.

The encoder is the part of the network that compresses the input in a space of latent variables, and which can be represented by the encoding function h = f (x). The decoder is the part that deals with reconstructing the input based on the information previously collected. It is represented by the decoding function r = g (h). The use of an autoencoder in this P300 detector architecture lies in the fact that training the autoencoder to copy the input, the space of latent variables h (code) can take on useful characteristics. This can be achieved by imposing limits on the coding action, forcing the space h to dimensions smaller than those of x. In this case, the autoencoder is known as undercomplete. By training the undercomplete space, we bring the autoencoder to grasp the most relevant characteristics of the training data [32]. 

For this aim, the L_1_ of the autoencoder in Figure 4 consists of a slicing procedure, aiming to singularly scorporate the 6 channels. The resulting tensor shape is (16,) according to Tensorflow and Keras shape nomenclature. The encoder part is realized utilizing a fully connected layer L_2_ with 16 units activated by the ReLU function. The ReLU activation function has been largely used in this architecture because the efficient computation ReLu(x) = max(x,0) permits a faster convergence during training [33]. Moreover, used in deep networks, ReLU does not saturate, avoiding the vanishing gradients problem [34]. L_3_ serves as code layer and is composed of an 8-unit dense layer with ReLU activation. It allows one to compress the initial tensor extracting only meaningful features. L_4_ acts as a decoder and is a mirrored version of L_2_. The output layer, the L_5_, is a fully connected layer with the same size as the input tensor. It does not have an activation function to properly reconstruct the input in a more useful way. The autoencoder structure has been distinctly compiled with respect to the remaining architecture. Specifically, the model fit was based on a mean squared error (MSE) loss function, a root mean square propagation (RMSprop) optimizer and monitoring the mean absolute error (MAE) as a metric for the weights update. A minibatch size of 32 samples was selected for the application. The fit resulted in 2144 parameters properly freezing for the implementation in the overall architecture. Table 2 summarizes the autoencoder characteristics focusing on the distribution of the parameters. 

**Figure 5:**
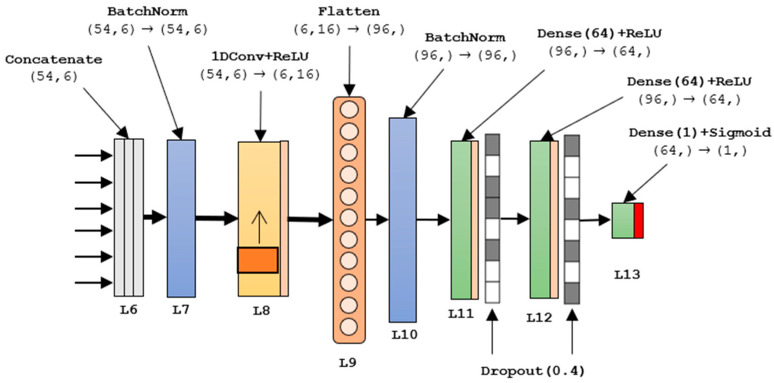
8-layer sequential NN implementation.

The tensors resulting from the parallel autoencoding were then concatenated along the 3rd dimension resulting in the initial size (54,6). The resulting tensor is then sent to the 8-layers CNN presented in Figure 5. Since the batch normalization step can reduce the covariate shift in neural network training, another batch normalization was added in the beginning of the CNN following the guidelines in [35]. It constitutes the L_7_.

The normalized tensor was sent to a 1D convolutional layer, the L_8_, for temporal feature extraction and subsampling. The convolutional kernels had size 16 × 1 with ReLU activation and a stride of 8 samples. For this reason, the time series was subsampled, reaching a length of only 6 samples. The resulting tensor of shape (6,16) was flattened by L_9_, realizing a (96,) size feature vector. The subsampling rate of 8 led to a temporal filter length of about 100 ms. Notably, the temporal convolution over L_8_ was not overlapped to save a lot of computational effort and avoid redundant features for the following layers.

The normalized vector from L_10_ went through 2 sequential dense layers, L_11_ and L_12_, with the same characteristics: 64 units and ReLU activation. Both the layers included a dropout stage with a dropout rate of 0.4 from a trial-and-error application. The dropout step was necessary to reduce coadaptions between neurons [36]. It forced the neuron to learn more robust features, limiting, in this way, the overfitting phenomenon. Only during the forward propagation training, with a dropout rate of 0.4, as in our application, every neuron of the layer has the 0.6 probability of being set to 0. During the test, every neuron is preserved. The outer layer, L_12_ consists of a single unit layer (binary classification) with a sigmoid activation function. This activation function returns a number in the range [0, 1] that represents the probability of a trial to be a P300 trials. Setting a threshold to 0.5 makes it possible to discriminate if there is a P300 or not. Table 3 summarizes the network characteristics, focusing on the distribution of the parameters. 

The compiling settings for the overall neural network training included a binary cross-entropy loss function and an Adam gradient descendent optimizer. Specifically, the Adam optimizer learning rate was set to 10^−4^ with no decay, while its parameters β_1_, β_2_, and *ε* were set, respectively, to 0.9, 0.999, and 10^−8^ as per [37]. The batch size for the stochastic gradient descendent was set to 64.

The proposed model has a total of 14,857 parameters, of which only 12,477 have been trained, since 2380 came from the freezed autoencoder section.

The whole neural network described above was realized in Python by using the Keras library. Next, the model was then saved and imported in the STM32CubeMX environment for code migration (Python → C), quantization cross-check, and on-board validation.

### 2.6. Output Management

The outputs provided by the classifier were managed differently for each analyzed dataset. Considering Dataset 1, each selection of direction to be taken consisted of 2 directions scintillations. Ideally, every 8 flashes (2 per each target), the decision was taken as the target that returns the maximum occurrence. Eight flashes corresponded to a decision every 1.4 s considering an ISI of 175 ms. 

In similar way, the flashing of 6 rows and 6 columns in the experiment was repeated 15 times per character epoch [8]. Initially, this procedure was chosen because the P300 detection in a single trial over 36 characters was very low. The probability values corresponding to multiple row/column flashing could then be accumulated (repetitions number). Thus, the target character can be defined as that character that corresponds to the row and column with the highest occurrence value. 

## 3. Results

Section 3.1 is dedicated to the system performance extraction with respect to both the datasets. For this purpose, four metrics are monitored: precision, recall, binary accuracy ad F1 score as defined in Equation (1). Section 3.2 analyzes the BCI performance in terms of character recognition accuracy providing comparison graphs with respect to the state of the art. In Section 3.3, the ITR is analyzed for both the proposed applications, in order to evaluate the system speed. Section 3.4 outlines the LBP routine impact on the P300 detection accuracy metrics by considering several neural network architectures. Finally, Section 3.5 is dedicated to the architecture implementation on a dedicated microcontroller.

### 3.1. BCI Performance: Single-Trial Classification Metrics

The performance of the single-trial P300 detection is analyzed in the following by means of four metrics: precision, recall, binary accuracy ad F1 score as defined in Equation (2).

These metrics are defined as:

(4) $$\mathrm{Precision}\;\left(\%\right)=100*\frac{\mathrm{TP}}{\mathrm{TP}+\mathrm{FP}}$$

(5) $$\mathrm{Recall}\;\left(\%\right)=100*\frac{\mathrm{TP}}{\mathrm{TP}+\mathrm{TN}}$$

(6) $$\mathrm{Acc}.\;\left(\%\right)=100*\frac{\mathrm{TP}+\mathrm{TN}}{\mathrm{TP}+\mathrm{TN}+\mathrm{FP}+\mathrm{FN}}$$

where TP is the number of P300 trials correctly classified, TN is the number of non-P300 trials correctly classified, FP is the number of non-P300 trials misclassified (classified as P300 trials) and, similarly, FN is the number of P300 trials misclassified. 

Among the above-mentioned metrics, the F1-score is well-correlated with the direction or character recognition accuracy and, thus, generally considered the more reliable metrics for imbalanced datasets such as P300-based ones. The other metrics suffer from the imbalanced nature of the dataset [10].

To address the problem of imbalanced datasets, in this work, a mixed approach undersampling/oversampling was adopted. Data from both datasets underwent a first stage of dataset undersampling based on the NearMiss version 2 from imblearn Python library and, secondarily, upsampling by the RandomOverSampler method from the same library [38].

For the sake of a coherent comparison, the same metrics were extracted from other state-of-the-art solutions which analyzed the same dataset: SVM-1 [13], CNN-1 [12], BN3 [10], ConvLSTM [11], their ensemble provided by [11], PCA-NN [22], the ERPENet [24]. Table 4 reports the comparison results over the BCI competition dataset (Dataset 2). Only the CNN-1 [12], BN3 [10], ConvLSTM [11], BN3+ConvLSTM [11] provided a complete overview of these metrics in their works. ERPENet by [24] performs a general average plus standard deviation value over both the subjects involved in the Dataset 2 composition (i.e., 83.54 ± 1.53). However, no explicit reference is reported with respect to the accuracy extraction modalities (e.g., validation or testing related accuracy). PCA-CNN [22] and SVM-1 [13] do not provide the investigated metrics. Moreover, Table 4 reports two versions of the here-proposed autoencoded CNN. A first version, named autoencoded CNN in Table 4, is the here-presented method in all of its steps, while the second version, i.e., autoencoded CNN no LBP, consists of the same model without the LBP preprocessing step but with an average based downsampling to match the LBP code length, leaving the number of the model parameters unaltered.

The system performance has been also validated on the prototype car driving dataset, where the decision is among four possible directions. Table 5 summarizes the results obtained on the single-trial P300 detection all over the four analyzed subjects.

**Figure 6:**
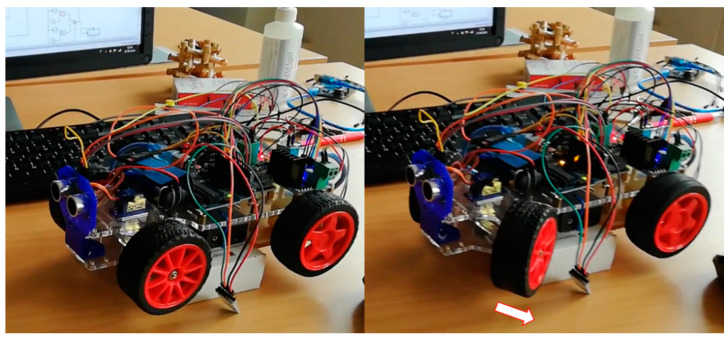
Snapshot of an in vivo proof of concept validation on the acrylic prototype car system designed in [3].

To provide a complete overview of the system workflow from the acquisition up to the mechatronic actuation, a snapshot from the in vivo proof of concept validation on the prototype car model designed in our previous work [3] is shown in Figure 6.

### 3.2. BCI Performance: Character Recognition Accuracy

**Figure 7:**
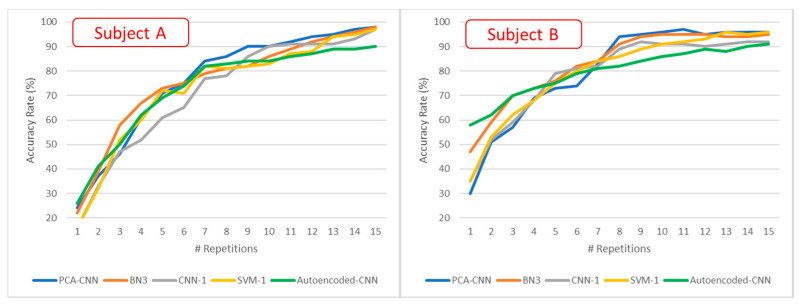
Accuracy rate on subjects A and B for Dataset 2.

**Figure 8:**
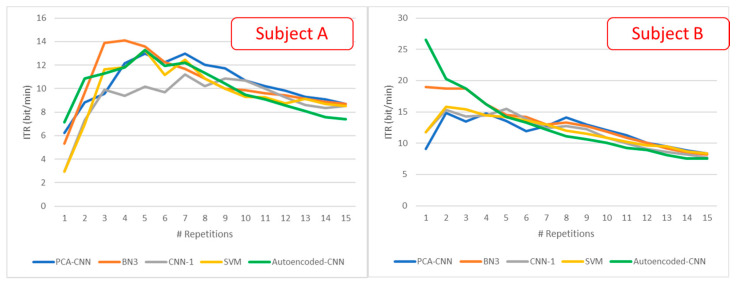
ITR graph for four algorithms on Dataset 2.

Figure 7 shows the accuracy rate in recognizing among the 100 test characters provided with Dataset 2 versus the number of considered repetitions. Figure 7 excludes from the comparison the ConvLSTM and the ensemble model, because no results are provided in the related articles [11]. It must be specified that no trial accumulation nor average have been carried out concerning the here-proposed method. The values reported in Figure 8 are fully extracted from a single-trial occurrence over all the repetitions according to the procedure reported in Section 2.6.

### 3.3. BCI Performance: Information Translate Rate

Another metric widely used to characterize a BCI system in terms of speed is the information translate rate or ITR. This parameter provides an idea of the number of bit/minute that the BCI architecture can provide, taking into account the classification accuracy. As per [19,39], ITR can be defined as:

(7) $$\mathrm{ITR}=\frac{60(P*\log_{2}\left(P\right)+\left(1-P\right)\log_{2}\left(\frac{1-P}{N-1}\right)+\log_{2}\left(N\right))}{T}$$

where N represents the number of recognizable classes (N = 36 in the speller problem, N = 4 in the prototype car application), P represents the accuracy rate in character/direction recognition and T is the time needed for characters/directions recognition.

Concerning the T parameter, it assumes different values for the different recognition problems. Considering the recognition of the direction for the prototype car driving application, some considerations should be taken. Each direction scintillation lasts about 100 ms with a blank status of about 75 ms for a total of 4 directions* 175 ms × n_rep_ = 700 ms × n_rep_, with n_rep_ number of repetitions needed to provide the direction choice. A pause of 500 ms follows the selection in order to ensure a driving fluidity. Considering the above-mentioned timing constraints, the T value for this specific application can be extracted by:

(8) $$T=0.5+0.7{*n}_{\mathrm{rep}}\;{\mathrm{with}\;n}_{\mathrm{rep}}=\left\{1,2\right\}$$

In a similar way, rows/columns scintillation lasts about 100 ms interspersed by a 75 ms pause for a total of 12 among rows and columns* 175 ms × n_rep_ = 2100 ms × n_rep_, with n_rep_ number of repetitions needed to provide the character choice. A pause of 2.5 s follows the selection. Considering the above-mentioned timing constraints, the T value for this specific application can be extracted by:

(9) $$T=2.5+2.1{*n}_{\mathrm{rep}}\;{\mathrm{with}\;n}_{\mathrm{rep}}=\left\{1\ldots 15\right\}$$

Figure 8 shows the ITR achieved by the proposed algorithm and the above analyzed state-of-the-art solutions considering the accuracy rate from the Dataset 2.

Considering the Dataset 1, the ITR achieved by the autoencoded CNN on a single or on a double repetition to decide the prototype car direction is 32.44 bit/min and 28.35 bit/min, respectively.

### 3.4. Local Binary Patterning Impact on BCI Performance

To analyze the LBP impact on other neural network architectures used in the P300 recognition analysis context, the CNN-1 [12], BN3 [10], ConvLSTM [11], BN3+ConvLSTM [11] structure have been reproposed in Python by using the Keras library with Tensorflow backend [40]. The implemented architectures have been realized according to the details provided in the related works. Specifically, BN3 architecture has been implemented in two forms: (i) the original one, namely BN3 and (ii) a BN3 version without the first spatial filtering stage, named BN3(ns) in the following. The above-mentioned architectures have been tested on the 6-channels EEG setup defined by the recursive channel elimination procedure provided in Section 2.3. Results concerning the LBP impact on the BCI performance are shown in Table 6.

### 3.5. Microcontroller Implementation

One of the main focuses of the present paper has concerned the full compatibility of the NN in a microcontroller implementation context.

**Figure 9:**
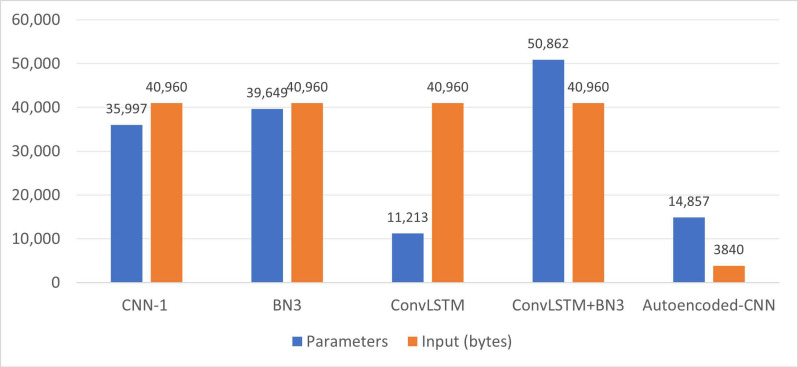
Histogram plot of the number of NN model parameters and input data size (bytes) for the analyzed methods.

For this aim, a first important comparison that should be carried out before starting the implementation analysis, concerns the number of total parameters composing each model analyzed up to now, as well as the amount of data request in input to permit the model working properly. Figure 9 summarizes these parameters via histogram view, comparing only those above-introduced methods that provided these metrics in their works [10,11,12].

For the complexity analysis, the proposed model was realized in Python by using the Keras library with Tensorflow backend [40]. Next, the different NNs composing the final autoencoded-CNN architecture were extracted as separate *.h5* models for the C-code generation and combination. The target hardware chosen for validation purposes is the Nucleo board L476RG.

For this aim, the Nucleo board L476RG was instantiated by using the STM32CubeMX peripheral initializer [41]. The STM32CubeMX extension, i.e., X-CUBE-AI, was used to integrate the NN model for a real-time inference assessment. This package provides an automatic and advanced NN mapping tool to generate and deploy optimized and robust C-model implementation of a pretrained NN for embedded systems with limited and constrained hardware resources. This package was chosen because of the possibility of obtaining post-training quantization support for the Keras models, which allows the user to check the error related to the quantization operated to translate the Keras model in a C one.

**Figure 10:**
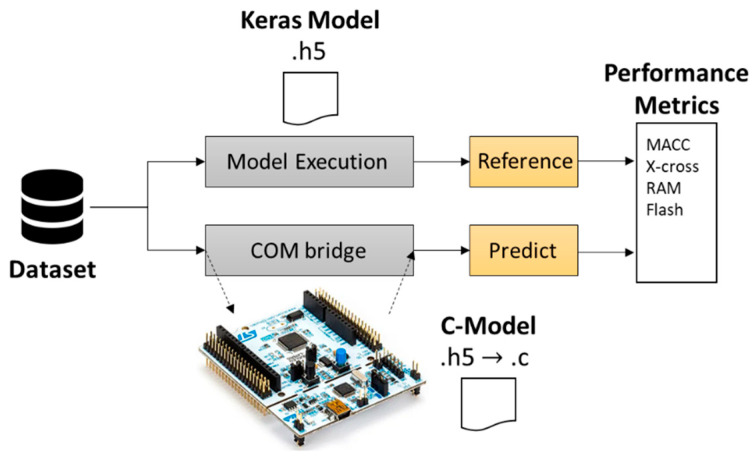
Validation on target flow by X-CUBE-AI.

A simple validation mechanism was implemented to check the accuracy of a C-generated method and the uploaded pretrained model from Keras. The validation engine procedure is schematized in Figure 10. It consists of sending a preprocessed dataset (or a random number generator with mean and standard deviation from the input dataset) in two dedicated branches: (i) the original model framework and (ii) a serial bridge toward the Nucleo board that runs the translated C-model. Next, the labels from the model execution block are used as a reference to be compared with the prediction from the COM bridge. The comparison is then used to provide several complexity metrics. The metrics analyzed in this section are RAM that indicates the size (in bytes) of the expected R/W memory used to store the intermediate inference computing values and ROM/Flash, indicating the size of the generated read only memory to store weight and bias parameters. Finally, the complexity metric is provided by the Multiply and ACCumulate (MACC) operations that universally define the functional complexity of the imported NN.

**Figure 11:**
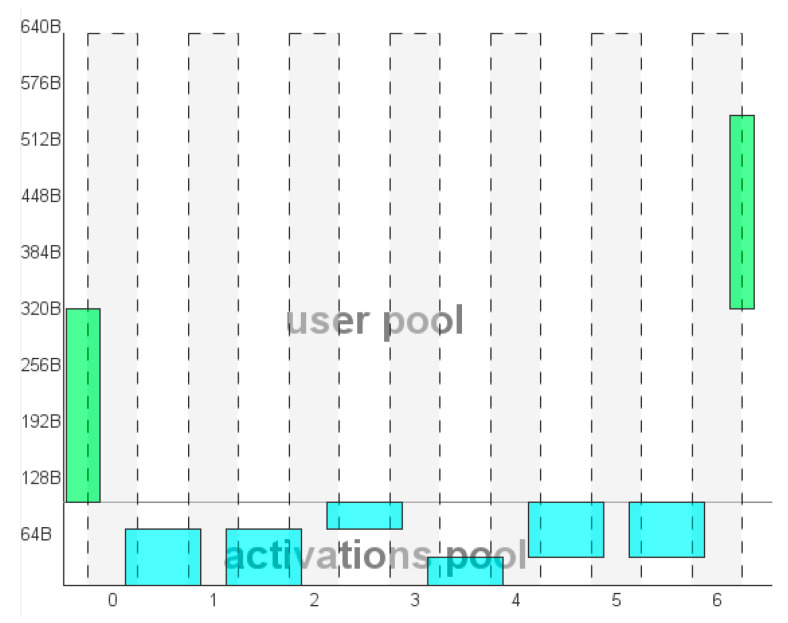
RAM and I/O memory usage of the autoencoder stage.

As a first step, we evaluate the implementation metrics for the first autoencoder stage. Overall, it results in a complexity of 2184 MACC, a flash occupation of 8.38 kB over 1024 kB available and a RAM occupation of 544 B, comprising a single branch input and a single branch output vector (3.264 kB in total). RAM also comprises 96 B from activation. The MACC parameter provides an estimation of the autoencoder operative timing. Since with an STM32 Arm^®^ Cortex ^®^—M4, each MACC corresponds to about nine clock cycles and considering the system clock set to 80 MHz, the time needed to encode and decode the symbolization code is ~246 µs. Validated on target, this timing request resulted to be 307 µs. Details about the Flash utilization, MACC usage and validated timing per layer are provided in Table 7, while the RAM occupation is shown layer by layer in Figure 11. Finally, the quantization comparison returns an error on the prediction matching of 4.92 × 10^−7^, largely below the threshold of 0.01 that ensures a good model match regardless of the quantization.

**Figure 12:**
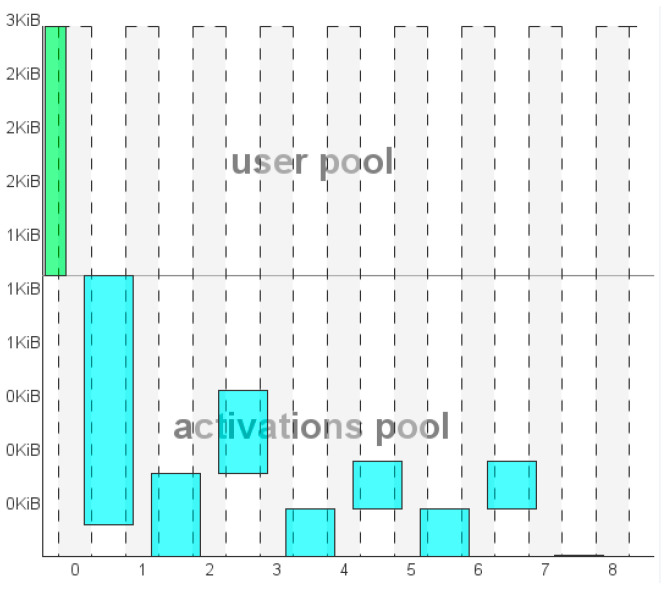
RAM and I/O memory usage of the sequential NN following the autoencoder.

As a second step, we evaluate the layers composing the remaining sequential structure. Overall, it results in a complexity of 17,995 MACC, a flash occupation of 48.74 kB over 1024 kB available and a RAM occupation of 2.79 kB for the I/O management. RAM also comprises 1.48 kB from activation layers. The MACC parameter allows one to estimate an operation timing of about ~2.02 ms @ 80 MHz. Validated on target, this timing request resulted to be 2.663 ms. Details about the Flash utilization, MACC usage, and validated timing per layer are provided in Table 8, while the RAM occupation is shown layer by layer in Figure 12. Finally, the quantization comparison returns an error on the prediction matching of 8.98 × 10^−7^, largely below the threshold of 0.01.

## 4. Discussion

The proposed single-trial P300 detector based on an autoencoded CNN-like architecture was designed with a first main goal of achieving the maximization of the ITR, while keeping high recognition accuracy. As second goal, we investigated the full implementability of the whole architecture on a low-cost dedicated microcontroller-based platform, the STM32L476RG Nucleo board, minimizing the resources utilization.

Concerning the single-trial P300 detection framework, the experimental results on the same dataset from the BCI competition III (i.e., Dataset 2), reported in Table 4, showed how on subject A, the proposed autoencoded CNN model ensures a precision comparable with the best model that is the ensemble one composed by BN3 and ConvLSTM. The best model in terms of recall results to be CNN-1; however, the autoencoded CNN model achieved a competitive recall (−0.14%) in the same application. The autoencoded CNN design had a focus on a metric that results in a tradeoff among precision and recall, the F1-score. Considering this metric, the autoencoded CNN ensures an F1 score of 47.37%, which is +0.67% with respect to the ensemble model. On subject B, the proposed model performs better than the other solutions in terms of precision, binary accuracy and F1 score, with +0.84%, +0.38% and +1.03%, respectively. The model that presents the best recall is the ConvLSTM.

**Figure 13:**
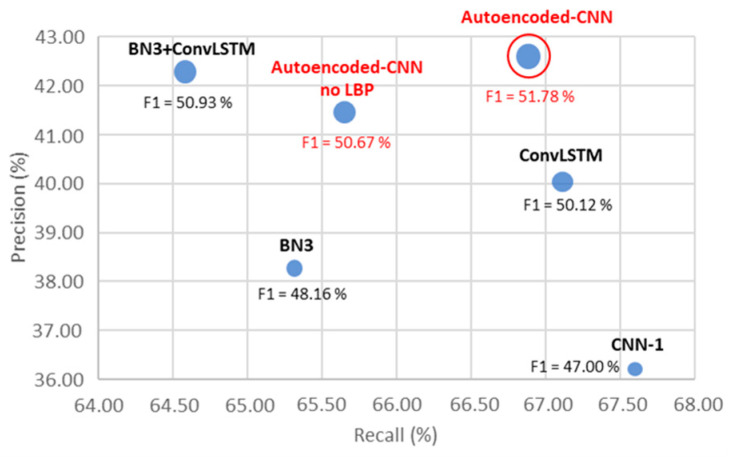
Precision versus recall versus F1-score bubble scatter plot for model comparison.

To provide a complete overview of the multi-objective optimization achieved by the proposed method, a bubble scatter plot reporting precision on *y*-axis, recall on *x*-axis and F1-score on the bubble dimension is presented in Figure 13. All the parameters reported in Figure 13 are extracted by averaging values from subjects A and B from Dataset 2.

The red model names are related to the here-presented one as per the definition in Table 4. Considering the scatter plot’s nature, the best single-trial P300 detector system should lie in the top-right part of the plot, which means precision and recall are maximized. The bubble should also be as large as possible to maximize the F1-score.

The scatter plot in Figure 13 shows how the autoencoded CNN outperforms on average the other state-of-the-art solutions in terms of precision and F1-score, placing itself in the highest top-right corner of the comparison plane. It demonstrates the capability of the model in proper weighting imbalanced datasets such as P300-based ones, where the target trials are largely below the not target ones (target: 16.67% of the total dataset, not target: 83.33%). Moreover, results concerning (Table 5) the single-trial P300 recognition in a prototype car driving experiment showed that the system performs better in a four-choice BCI paradigm than a 36-character P300 speller [42,43]. Indeed, with an average precision of 68.83 ± 8.86%, the system overcomes its own performance on the P300 speller problem by about + 26.23%. In a similar way, an improvement of +4.87% has been recorded (71.75 ± 2.10% versus 66.88% from P300 speller) in recall metric and +5.54% in the binary accuracy context (84.45 ± 3.53% versus 78.91% from P300 speller). Finally, the F1-score also returns an improvement of +18.22%, considering an average recall of 51.78% in the P300 speller problem and 70.00 ± 4.58% recorded in the prototype car driving problem. The F1-score improvement between the two datasets can be related to the lower number of choices in the four-choice odd ball paradigm (Dataset 1) with respect to the 36-choice paradigm of the P300 speller. Indeed, in the first case, the ratio between target and not target stimulations is 1:4, making the dataset “less unbalanced” than the P300 speller case (1:6), negatively influencing the harmonic mean between precision and recall [44]. Additionally, no explicit information has been provided by the Wadsworth Research Center NYS Department of Health for data collection routines at the international BCI competition III. EEG channels’ data have been provided with different SNRs, and a lot of outliers have been recorded on the Fz channels, which is one of the most informational ones according to the study conducted by authors in [22]. The data collection routine for the car prototype fluid driving has been—instead—conducted in a controlled environment, by regulating lights and minimizing distracting phenomena and with a real-time impedance check on each electrode. It could result in a performance improvement.

This paper also presented the character recognition rate results in Figure 7. The experiment conducted on the test set by Dataset 2 [21] showed how the proposed autoencoded CNN model performs better than the other solutions for a few row–column repetitions (considering the range going from 1 to 3 repetitions). Then, the number of FP starts to become critical, reducing the accuracy in the comparison. Overall, the autoencoded CNN is able to achieve an average character accuracy of the 42% after a single character repetition, overcoming by about +7.5% the second-best architecture, the BN3 one. After 15 repetitions, the accuracy reached, on average, is 90.5%, about −6.5% under the best model implemented by the joint application of PCA and CNN.

Next to these accuracy values, we analyzed another metric widely used to characterize a BCI system in terms of speed, the ITR, which constitutes one of the main constraints of this design. In this context, results in Figure 8 show that the autoencoded CNN ITR is able to reach on average 16.83 bit/min with a single repetition, performing better than other solutions. The ITR performance after eight repetitions degrades proportionally with the accuracy rate, losing—on average—1.64 bit/min if compared to the best solution realized via the BN3 network. Considering subject B, the system achieves an ITR of 26 bits/min that formally means one correctly brain digitized character every 2 s. In the same context, with an ITR of 32.44 bit/min, the user can formalize and actuate a direction choice about every 1.8 s.

Contextually, the paper also investigated the impact of the proposed LBP routine as the preprocessing stage for several state-of-the-art neural networks. Specifically, the CNN-1 [12], BN3 [10], ConvLSTM [11], BN3+ConvLSTM [11] structure have been reproposed and used as the final classification step, substituting the raw EEG signals from 64 channels with the six-channel EEG setup defined by the recursive channel elimination procedure (Section 2.3). The BN3 architecture has been reproposed in two forms: (i) the original one, BN3, and (ii) a BN3 version without the first spatial filtering stage (i.e., BN3(ns)).

**Figure 14:**
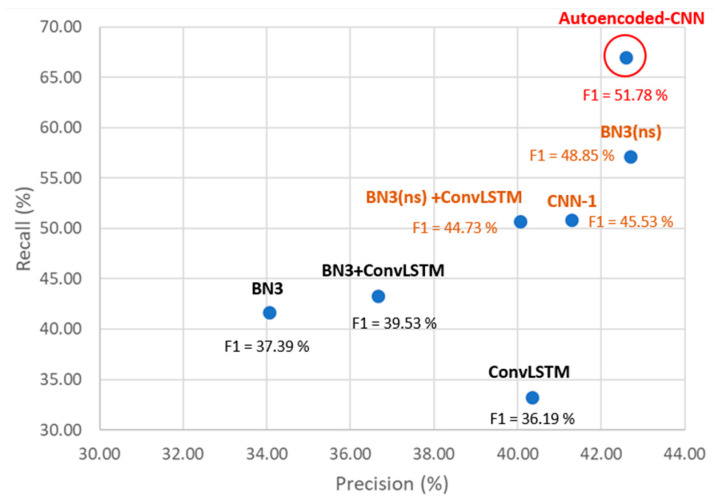
Precision versus recall scatter plot for LBP impact on neural network architecture comparison.

According to the results in Table 6, a scatter plot reporting precision on the *x*-axis and recall on the *y*-axis is depicted in Figure 14. As per Figure 13, all the parameters reported in Figure 14 are extracted by averaging values from subjects A and B from Dataset 2.

The red model names are related to the here-presented model as per the definition in Table 4 and Table 6.

Considering the scatter plot (Figure 14), the best single-trial P300 detector system should lie in the top-right part of the plot, which means precision and recall are maximized.

It is interesting to note that the LBP routine works better as a preprocessing stage for those neural networks that implement a first 1D convolutional stage dedicated to the temporal feature extraction (temporal convolution), as highlighted by the brown colored models in Figure 14. For instance, by using the LBP as a preprocessing stage, the BN3 architecture without a spatial filtering layer (i.e., BN3(ns)) performs better (+7.46% on F1-score) than its version with a first stage of spatial filtering (i.e., BN3). The problem with these CNNs with the LBP routine seems to lie in the presence of the first spatial convolutional operation that converts each column of spatial data from the input tensor in an abstract feature map. This feature map is—thus—sent to a temporal convolution layer that analyzes—in this way—an abstract temporal signal rather than a raw temporal one. This problem has been also analyzed by the authors in [45], which demonstrated that the introduction of a spatial convolution layer as a first stage in a P300 detector application strongly increased the following CNN complexity to achieve good results. Since most of the main P300 features have a temporal nature, the presence of this spatial filtering degrades the LBP capabilities. The introduction of an autoencoder between the LBP and the first temporal convolution stage also improves the neural network’s performance, leading to a +4.21% on F1-score for the BN3(ns), +5.31% for the ensemble BN3(ns)+ConvLSTM and a +1.24% for the CNN-1. The reason lies in the capabilities of the encoder–decoder process to denoise the signal, while preserving the useful temporal information [32].

Beyond the ITR-accuracy tradeoff, the second main design goal for the here-presented system was the architecture implementability on a low-cost microcontroller or dedicated embedded platform. For this reason, Figure 9 summarizes the number of implemented parameters and the memory usage for those above-introduced methods that provided these metrics in their studies [10,11,12]. Results in Figure 9 show how with a total of 14,857 parameters, the here-proposed autoencoded CNN lies largely below most of the state-of-the-art solutions (about 21,140 parameters less than the CNN-1). Only the ConvLSTM by [11], with 11,213 parameters, slightly reduces the neural network complexity (−3644 parameters) with respect to the here-proposed method; however, it requires the parallel analysis of trials from 64 EEG channels strongly increasing the input data size (+37.12 kB w.r.t. the autoencoded CNN). It must be specified that all the compared models exploit a high amount of data as input. Indeed, all of them consider data from all the 64 channels and different trial lengths against a monitoring of only six channels proposed in the present work. Nevertheless, assessing 64 channels in parallel with a minimum sampling rate of 240 Sa/s is not practically applicable in mobile EEG applications equipped with microcontrollers due to the overflow of RAM capabilities. For this aim, the present work uses only six properly selected channels, which strongly reduce the memory resources consumption. Considering this first complexity view and accuracy results, we can infer that the autoencoded CNN provides the best tradeoff between accuracy, speed, and complexity.

Concerning the full architecture implementation, the autoencoded CNN was implemented and validated on a Nucleo board L476RG placed in a COM bridge and in the inference results with the realized Keras model. Overall, the whole system (Autoencoder + CNN) results in a complexity of 20,179 MACC, a flash occupation of 57.11 kB over 1024 kB available and a RAM occupation of 2.85 kB over 96 kB available on the board.

The MACC parameter allows one to estimate an operation timing for the overall inference at about ~2.27 ms @ 80 MHz. Validated on target, this timing request resulted to be 3.175 ms. The quantization comparison returns an error on the prediction matching of 4.61 × 10^−6^, below the threshold of 0.01.

It must be also specified that the here=proposed method, with a minimum flash request of 57.11 kB and a minimum RAM of 2.85 kB (without compression process), resulted to be easily implementable on all the STM32 microcontrollers family above the F3 one. It leads to a low-cost solution for the realization of a potentially dedicated embedded platform if we consider—for the sake of example—the use of an STM32F301C8 microcontroller with a cost of USD 0.16 as a central computation core. The use of this low-cost microcontroller ensures all the needed memory capabilities without any additive external memories need. The proposed microcontroller also includes several communication protocols to interface low-cost WiFi dongles such as an ESP8266 to interface the wireless EEG headset.

## 5. Conclusions

In this paper, a single-trial P300 detector that combines a novel pre-processing stage based on the EEG signals symbolization and an autoencoded convolutional neural network (CNN), maximizing the accuracy in single-trial P300 detection, for a low number of stimuli repetition, enhancing—as a consequence—the BCI speed in terms ITR and ensuring the full compatibility with microcontrollers implementation, has been presented. Specifically, the proposed system exploited data from a small number of selected EEG channels to address the memory consumption issue. Next, a preprocessing routine which includes: (i) baseline correction, (ii) windsorizing and (iii) LBP symbolization was implemented and studied. The symbolized EEG signals were then sent to an autoencoder model to emphasize those temporal features that can be well comprised by the following sequential network composed of seven layers. This latter sequential structure is made up of a combination of a 1D convolutional layer for the temporal filtering, three fully connected layers, batch normalization and dropout stages. The method was tested on two different datasets: one from a P300 speller application in BCI competition III data and one from self-collected data implementing a fluid prototype car driving. The experimental results showed that, in a P300-speller application, the proposed method was able to outperform the state of the art in terms of F1-Score = 51.78 ± 6.24%, ensuring a +5 bits/min of average ITR if compared with other state-of-the-art solutions. The same method, applied to the prototype car driving experiment, provided an F1-score of 70.00% and an ITR > 30 bit/min, allowing a car direction change every 1.8 s. The implementation feasibility assessment on an STM32L4 microcontroller showed that the overall system occupies 5.57% of the total available ROM, ~3% of the available RAM. Considering this complexity assessment, the ITR and accuracy results, it can be inferenced that the autoencoded CNN provides a good tradeoff between accuracy, speed, and complexity to realize a single-trial P300 detector. Moreover, an example of design conceptualization has also been proposed and discussed, demonstrating the P300 detector implementability on small footprint and low-cost dedicated microcontroller-based boards. In a real-life scenarios application context, the here-proposed P300 detector was demonstrated to be able to successfully expand the capabilities of autonomous driving systems, while its application in brain-controlled assistive robotics provided promising initial results.

## Figures and Tables

**Table 1:** Training and test datasets composition.

Dataset	Sub.	Training Set ^1^		Test Set ^1^	
		P300	non-P300	P300	non-P300
Dataset 1	1…4	1520	4560	500	1500
Dataset 2	A, B	2550	12,750	3000	15,000

^1^ Data expressed in trials per subject.

**Table 2:** Autoencoder layers’ characteristics.

Layer	Input	Type	Output	# Parameters
L_1_	(54,6)	tf.Slice	(54,)	0
L_2_	(54,)	Dense(16)+ReLU	(16,)	912
L_3_	(16,)	Dense(8)+ReLU	(8,)	136
L_4_	(8,)	Dense(16)+ReLU	(16,)	144
L_5_	(16,)	Dense(54)	(54,1)	952

**Table 3:** Autoencoded CNN Layers’ characteristics.

Layer	Input	Type	Output	# Parameters
L_6_	(54,6)	Concatenate	(54,6)	0
L_7_	(54,6)	BatchNormalization	(54,6)	24
L_8_	(54,6)	Conv1D+ReLU	(6,16)	784
L_9_	(6,16)	Flatten	(96,)	0
L_10_	(96,)	BatchNormalization	(96,)	448
L_11_	(96,)	Dense(64)+ReLU	(64,)	7232
D_out1_		Dropout (0.4)		0
L_12_	(64,)	Dense(64)+ReLU	(64,)	4160
D_out2_		Dropout (0.4)		0
L_13_	(64,)	Dense(1)+Sigmoid	(1,)	65

**Table 4:** Performance of different models on test dataset from Dataset 2.

Subject		Model	TP	FP	TN	FN	Prec. (%)	Recall (%)	Acc. (%)	F1-Score (%)
A	**Our Work**	**Autoencoded CNN**	2017	3499	11,501	983	**36.57**	**67.23**	**75.10**	**47.37**
		**Autoencoded CNN no LBP**	1951	3419	11,581	1049	**36.33**	65.03	**75.18**	46.62
	State-of-the-Art Solutions	BN3	1910	3771	11,229	1090	33.62	63.67	72.99	44.00
		ConvLSTM	1928	3418	11,582	1072	36.06	64.26	**75.05**	46.20
		CNN-1	2021	4355	10,645	979	31.70	**67.37**	70.37	43.11
		BN3+ConvLSTM	1919	3299	11,701	1081	**36.78**	63.97	**75.67**	46.70
		ERPENet	Data N.A.						83.54 *	Data N.A.
		PCA-CNN	Data Not Available (N.A.)							
		SVM-1	Data N.A.							
B	**Our Work**	**Autoencoded CNN**	1996	2108	12,892	1004	**48.64**	66.53	**82.71**	**56.19**
		**Autoencoded CNN no LBP**	1988	2278	12,722	1012	46.60	66.27	81.72	54.72
	State-of-the-Art Solutions	BN3	2009	2671	12,329	991	42.93	66.97	79.66	52.32
		ConvLSTM	2099	2670	12,330	901	44.01	**69.97**	80.16	54.04
		CNN-1	2035	2961	12,039	965	40.73	67.83	78.19	50.90
		BN3+ConvLSTM	1956	2136	12,864	1044	47.80	65.20	**82.33**	**55.16**
		ERPENet	Data N.A.						83.54 *	Data N.A.
		PCA-CNN	Data N.A.							
		SVM-1	Data N.A.							

* The work reports only the average value of accuracy considering both the subjects.

**Table 5:** Performance on test dataset from Dataset 1.

Subject	TP	FP	TN	FN	Prec.	Recall	Acc.	F1-Score
1	364	269	1231	136	0.5750	0.7280	0.7975	0.6425
2	351	176	1324	149	0.6660	0.7020	0.8375	0.6835
3	349	99	1401	151	0.7790	0.6980	0.8750	0.7363
4	364	135	1365	129	0.7332	0.7420	0.8680	0.7376

**Table 6:** LBP impact on different state-of-the-art neural network architectures.

Subject		Model	TP	FP	TN	FN	Prec. (%)	Recall (%)	Acc. (%)	F1-Score (%)
A	**Our Work**	**Autoencoded CNN**	2017	3499	11,501	983	36.57	**67.23**	75.10	**47.37**
	State-of-the-Art Solutions	BN3	1096	3013	11,987	1904	26.67	36.53	72.68	30.83
		BN3(ns)	1611	2462	12,538	1389	39.55	53.70	**78.61**	45.55
		ConvLSTM	956	1998	13,002	2044	32.36	31.87	77.54	32.11
		CNN-1	1312	2144	12,856	1688	37.96	43.73	**78.71**	40.64
		BN3+ConvLSTM	1496	2464	12,536	1504	37.78	49.87	77.96	42.99
		BN3(ns)+ConvLSTM	1515	2406	12,594	1485	**38.64**	50.50	**78.38**	43.78
B	**Our Work**	**Autoencoded CNN**	1996	2108	12,892	1004	**48.64**	**66.53**	**82.71**	**56.19**
	State-of-the-Art Solutions	BN3	1401	1975	13,025	1599	41.50	46.70	80.14	43.95
		BN3(ns)	1812	2139	12,861	1188	45.86	60.40	81.52	52.14
		ConvLSTM	1035	1105	13,895	1965	**48.36**	34.50	**82.94**	40.27
		CNN-1	1735	2149	12,851	1265	44.67	57.83	81.03	50.41
		BN3+ConvLSTM	1098	1989	13,011	1902	35.57	36.60	78.38	36.08
		BN3(ns)+ConvLSTM	1523	2145	12,855	1477	41.52	50.77	79.88	45.68

* The work reports only the average value of accuracy considering both the subjects.

**Table 7:** Autoencoder layers’ ROM utilization and MACC.

Layer	Type	Flash (B)	MACC	Timing (ms)
L_2,1_	Dense(16)	3648	912	0.113
L_2,2_	ReLU	-	16	0.007
L_3,1_	Dense(8)	544	136	0.024
L_3,2_	ReLU	-	8	0.005
L_4,1_	Dense(16)	576	144	0.028
L_4,2_	ReLU	-	16	0.007
L_5_	Dense(54)	3808	952	0.124

**Table 8:** CNN layers’ ROM utilization and MACC.

Layer	Type	Flash (B)	MACC	Timing (ms)
L_7_	BatchNormalization	48	672	0.067
L_8_	Conv1D+ReLU	3136	5504	1.231
L_10_	BatchNormalization	896	224	0.028
L_11,1_	Dense(64)	28,928	7232	0.808
L_11,2_	ReLU	-	64	0.016
L_12,1_	Dense(64)	16,640	4160	0.746
L_13,2_	ReLU	-	64	0.016
L_13,1_	Dense(1)	260	65	0.014
L_13,2_	Sigmoid	-	10	0.008
